# Diet induced changes in the microbiota and cell composition of rabbit gut associated lymphoid tissue (GALT)

**DOI:** 10.1038/s41598-018-32484-1

**Published:** 2018-09-20

**Authors:** Rakel Arrazuria, Valentín Pérez, Elena Molina, Ramón A. Juste, Ehsan Khafipour, Natalia Elguezabal

**Affiliations:** 1 Department of Animal Health, NEIKER-Instituto Vasco de Investigación y Desarrollo Agrario, Derio, Bizkaia Spain; 2 Department of Animal Health, Faculty of Veterinary Medicine, University of Leon, Leon, Spain; 3 Department of Animal Science, University of Manitoba, Winnipeg, MB Canada; 4 Department of Medical Microbiology, University of Manitoba, Winnipeg, MB Canada; 5 Present Address: SERIDA, Agri-food Research and Development Regional Service, Villaviciosa, Asturias Spain

## Abstract

The gut associated lymphoid tissue (GALT) is the largest immune organ of the body. Although the gut transient and mucosa-associated microbiota have been largely studied, the microbiota that colonizes the GALT has received less attention. The gut microbiome plays an important role in competitive exclusion of pathogens and in development and maturation of immunity. Diet is a key factor affecting the microbiota composition in the digestive tract. To investigate the relation between diet, microbiota and GALT, microbial and cell composition of vermiform appendix (VA) and sacculus rotundus (SR) were studied in two groups of New Zealand white rabbits on different diets. Diet shifted the lymphoid tissue microbiota affecting the presence and/or absence of certain taxa and their abundances. Immunohistochemistry revealed that a higher fibre content diet resulted in M cell hyperplasia and an increase of recently recruited macrophages, whereas T-cell levels remained unaltered in animals on both high fibre and standard diets. These findings indicate that diet has an impact on the microbiota and cell composition of the GALT, which could act as an important microbial recognition site where interactions with beneficial bacteria can take place favouring microbiota replacement after digestive dysregulations.

## Introduction

The gut associated lymphoid tissue (GALT) is the largest immune organ of the body. It is a well-developed component of the mucosal immune system that is involved in protection of the host against pathogens and in the postnatal immune system maturation^1^.

In rabbits the structure of the GALT is even more developed than in other mammalian species^2^. The rabbit possesses two organized lymphoid tissue differentiated segments or organs: the sacculus rotundus (SR), which is located at the distal end of the ileum, forming the ileo-cecal junction, and the vermiform appendix (VA), located at the end of the cecum and that is considered an immune structure primarily functioning as a safe-house for beneficial bacteria^3^. These two lymphoid organs account for more than 50% of the total lymphoid tissue in the rabbit^4^.

The gut microbiota plays an important role in the development and maturation of intestinal mucosal immunity^5^ and contributes to the health of the host by colonizing the mucosal entry sites of pathogens. Moreover, the microbiota mediates resistance to infection indirectly by stimulating the innate immune response^6^. Many studies have documented differences in the composition of host associated microbial communities between healthy and diseased states^7,8^. It is recognized that an altered microbiome is not just a marker of disease but that it also actively contributes to pathogenesis^9^. According to the current knowledge on the cecal appendix function as ‘a “safe-house” for beneficial bacteria with the capacity to re-inoculate the gut following depletion of the normal flora after diarrheal illness’^3^, its microbiota has an even more important role in the health of the individual.

In the last decade, the great development of next generation sequencing technologies, has enabled researchers to investigate the digestive microbial composition under different conditions. However, although the gut transient and mucosa-associated microbiota have been largely studied, little is known about the microbiota that specifically colonizes the GALT which is directly interacting with the immune system. It has been demonstrated that specific microbial profile on vermiform appendix can induce inflammation^10^.

Diet has a major impact on health and it could be used in the near future as an alternative approach to control inflammatory and autoimmune diseases^11^. Diet is also one of the key factors affecting the composition of the microbiota in the digestive tract^12^ since dietary nutrients are the principal substrates for the microbial populations. Finally, diet can also have a direct effect on the immune response since food components beyond their function as nutrients, can play an important role in the operation of the immune system in health and disease^13^.

In the last years, many studies have documented the benefits of high fibre diets on human health, based on the production of short-chain fatty acids (SCFA) by the microbiota as a consequence of fibre degradation^14^. The predominant SCFAs are known to reduce the production of pro-inflammatory cytokines^15^ and recruit Treg cells as well as induce the expression of antimicrobial peptides^16^. Fibre based diets, have been shown to promote and increase gut microbiota diversity^17^ and also to diminish inflammatory responses by a mechanism that includes shaping the intestinal microbiome and indirectly affecting the immune system^18^. Moreover, a dietary fibre-deprived gut microbiota degrades the colonic mucus barrier and enhances pathogen susceptibility^19^. The effect of diet in experimental infection models in mice^20^ and rabbits^21^ has been studied, highlighting the importance of diet in experimental trials involving animals.

In addition to the diffuse lymphoid tissue formed by infiltrating cells interspersed at different densities throughout the regular mucosa, the GALT is formed by highly organized structures enclosed in the mucosal layer of the intestinal wall. These structures are formed by a lymphoid follicle covered by a dome of epithelial cells. Most of these epithelial cells are those known as M (microfold or membranous) cells. M cells play an important role in the transport of antigens from the lumen of the intestine to mucosal lymphoid tissues in which the processing and initiation of immune responses occur^22^. Antigens acquired by M cells are rapidly shuttled via vesicular transport to the basolateral membrane where they are released, enabling uptake by antigen presenting cells and processing for presentation to T cells^23^. Recently, it has been discovered that M cells have a critical role in the establishment of normal secretory immunity at GALT sites^23^. Moreover, it has been reported that dietary fibre and starch levels are related to the size and number of M cells in rabbit lymphoid tissue^24^.

In the rabbit, approximately 50% of the overlying epithelial cells are M cells whereas in in rats and humans only 5–10% of them are M cells^2^. The biological significance of this difference remains unknown. Recent studies focused on the characterization of rabbit GALT have concluded that rabbits are comparable to humans throughout their GALT supporting the use of the rabbit model to study human gut-associated disease or orally acquired infectious agents^25^. Moreover, SR and VA are highly differentiated areas that may be the reservoir from which microbiota replacement would start after digestive dysregulations^3^.

Diet, microbiota and immunity are highly connected, but our understanding of how this network functions is still limited. Because dietary fibre intake has demonstrated health benefits, the objective of this study was to assess the effect of a high fibre diet on microbiota and cell composition using the rabbit GALT model.

## Results

### Growth performance

Animal weight (Supplementary Fig. S2 and Table S2a) of Diet group A was significantly higher at the first three timepoints (weeks 0, 7 and 11). Weight increase among groups across different intervals (Supplementary Table 2b) did not show significant differences except for intervals 20–24 and 20–27 weeks where Diet B animals showed lower weight increase. Total weight increase (31–0 week) among groups did not show significant differences.

### Microbiota analysis

After quality control and removal of chimeric reads, an average of 22,657 (SD = 9,055) high quality sequences were obtained for downstream analyses. The rarefaction curve built with the observed species (Richness) showed asymptotic tendency (Supplementary Fig. S3), which indicates that the sampling effort was sufficient to compare species numbers.

**Figure 1:**
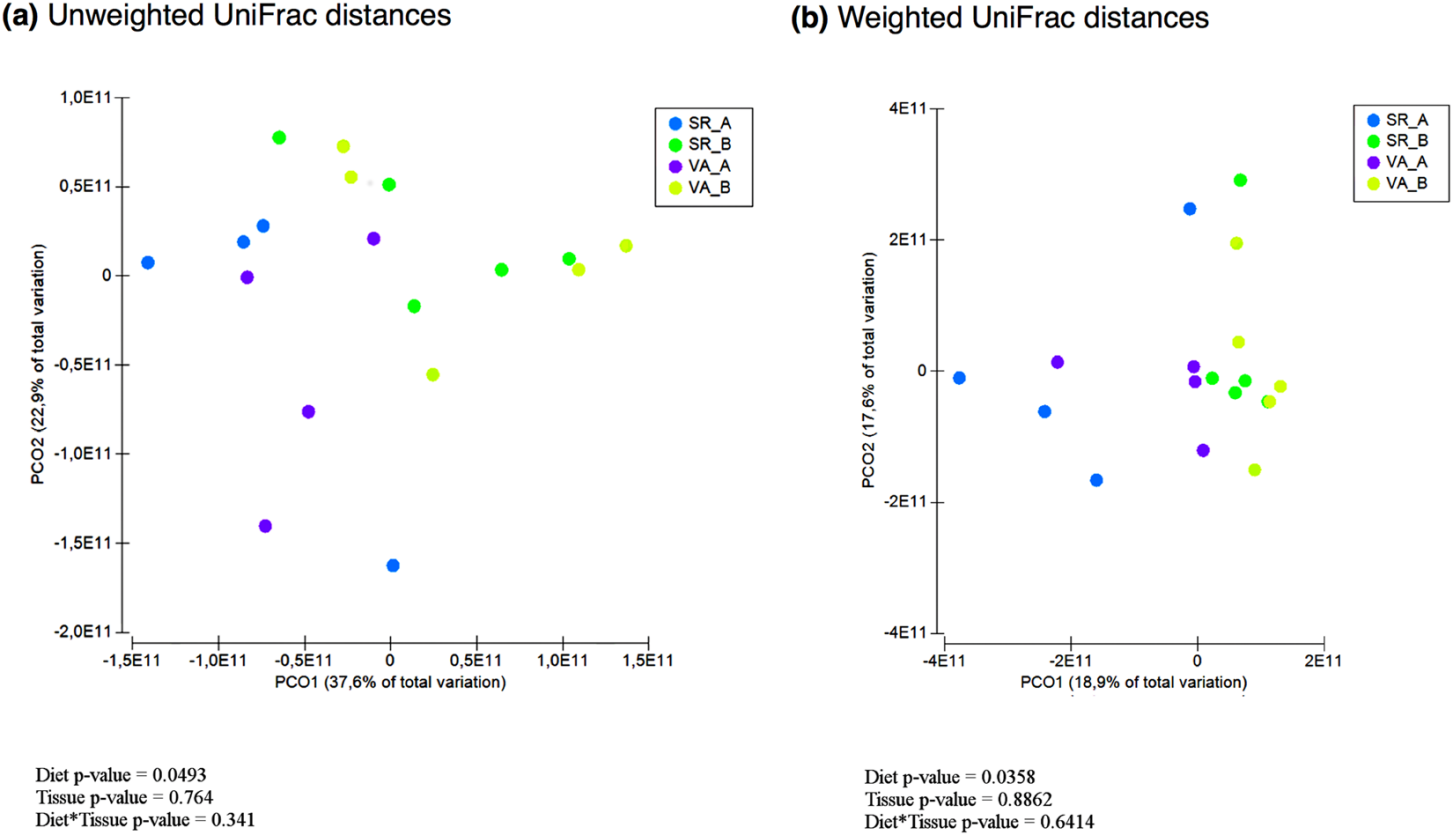
Principal Coordinates Analysis (PCoA) of the weighted **(a)** and unweighted **(b)** UniFrac distances. SR_A = Sacculus Rotundus Diet A; SR_B = Sacculus Rotundus Diet B; VA_A = Vermiform Appendix Diet A; VA_B = Vermiform Appendix Diet B.

Diversity analysis of the SR and VA samples revealed no significant differences in richness (Chao 1 index), or other alpha-diversity indices (Shannon and Simpson) between animals on diet A and B (Supplementary Fig. S4). The beta-diversity analysis of UniFrac distances revealed distinct clustering pattern between animals on diet A and B in both weighted (p = 0.049) and unweighted (p = 0.036) measures (Fig. 1a,b). However, when the distances between SR and VA were analyzed, no significant differences were observed in weighted (p = 0.764) and unweighted (p = 0.886) measures. Moreover, no interactions were detected between sampled tissue and diet when weighted (p = 0.341) and unweighted (p = 0.6414) distances measures were analyzed (Fig. 1a,b).

**Figure 2:**
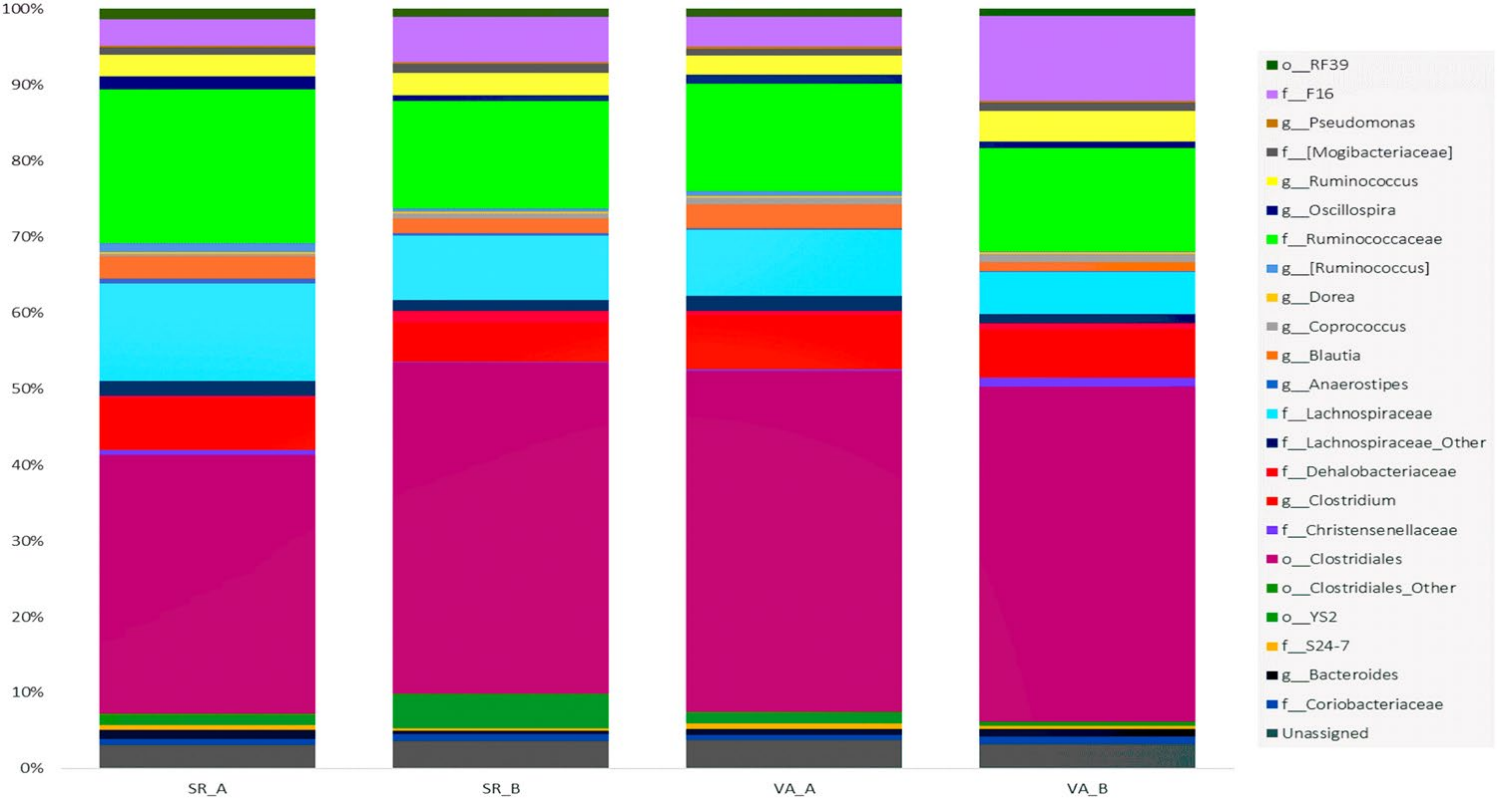
Stack-bar of the most abundant OTUs in Sacculus Rotundus (SR) and Vermiform Appendix (VA) on different diet (A,B). Vertical bars demonstrate the proportion (%) of the most abundant bacterial taxa (>0.1% of community). Taxonomic classification of each taxon is identified at phylum (p_), class (c_), order (o_), family (f_), and genus (g_) level.

Alignment of OTUs at 97% similarity threshold against Silva database resulted in identification of 7 bacterial phyla (Table 1) and 59 bacterial genera. While the majority of OTUs were identified at the genus (g.) level, some were only classified at the phylum (p.), class (c.), order (o.), or family (f.). The composition of the OTUs at the genus level in the four groups is presented in Fig. 2. The SR and VA microbiota was dominated by members of phylum Firmicutes followed by members of phylum TM7. Among all phyla, only the abundance of Bacteroidetes significantly declined in the SR of animals submitted to a diet B (p = 0.008; Table 1). At a deeper taxonomic level, the order Clostridiales was the most abundant one, representing the 38–43% of the observed taxa. The second more abundant taxon was the family Ruminococcaceae (13–16%) followed by the family Lachnospiraceae (7–10%), genus Clostridium (5–6%) and family F16 (4–7%). The remaining taxonomic groups were present in a relative abundance lower than 5% (Fig. 2).

**Table 1:** Average percentages of bacteria phyla in the sacculus rotundus and vermiform appendix on different diets and comparison analysis results.

Phyla	Mean values, %, on indicated conditions (SEM^a^)				p-values	
	Treatments^b^				SR.A vs. SR.B	VA.A vs. VA.B
	SR.A	SR.B	VA.A	VA.B		
Firmicutes	87.11 (5.28)	82.51 (3.60)	87.01 (3.65)	80.93 (3.38)	0.5123	0.2800
TM7	3.39 (1.76)	5.96 (1.62)	3.85 (1.70)	11.06 (2.90)	0.3318	0.0895
Bacteroidetes	2.10 (0.18)	0.80 (0.28)	1.63 (0.18)	1.76 (0.43)	0.0080^c^	0.7892
Cyanobacteria	1.25 (1.09)	4.35 (1.97)	1.35 (0.51)	0.52 (0.08)	0.2206	0.1122
Tenericutes	1.42 (1.07)	1.02 (0.38)	1.18 (0.78)	0.98 (0.16)	0.7534	0.8219
Actinobacteria	0.81 (0.34)	1.29 (0.20)	0.78 (0.17)	1.20 (0.13)	0.3003	0.1168
Proteobacteria	0.83 (0.63)	0.44 (0.15)	0.40 (0.07)	0.32 (0.09)	0.6051	0.5217
Unassigned	3.10 (0.75)	3.63 (0.58)	3.79 (0.79)	3.21 (0.40)	0.5997	0.5560

^a^SEM = Standard error of the mean. ^b^Treatments: SR.A = Sacculus Rotundus Diet A; SR.B = Sacculus Rotundus Diet B; VA.A = Vermiform Appendix Diet A; VA.B = Vermiform Appendix Diet B. ^c^p < 0.05 was considered significant.

**Figure 3:**
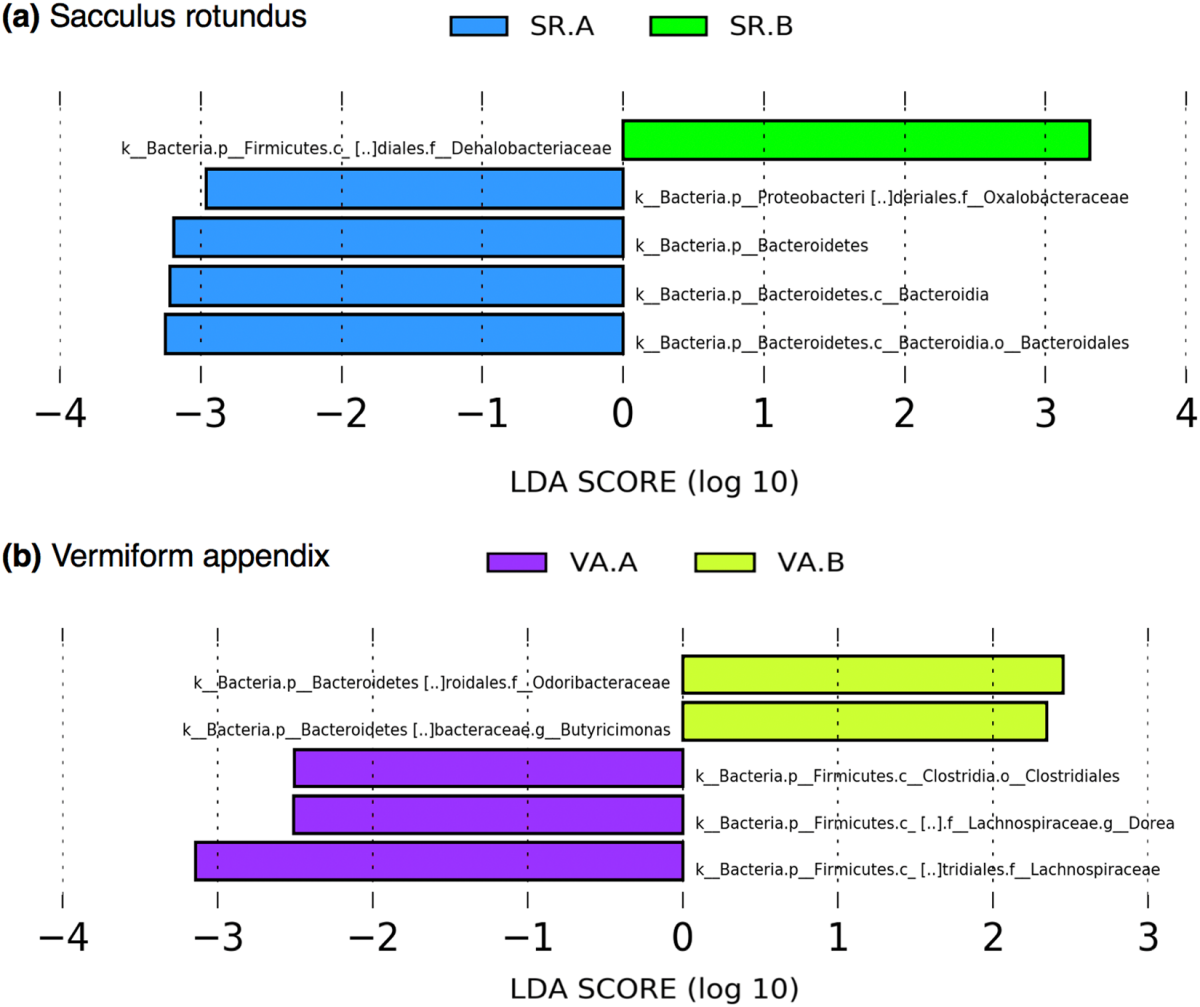
Differences in bacterial taxa related to diet in sacculus rotundus **(a)** and vermiform appendix **(b)** identified by linear discriminant analysis coupled with effect size (LEfSe) (LDA > 2, P < 0.05). SR.A = Sacculus Rotundus Diet A; SR.B = Sacculus Rotundus Diet B; VA.A = Vermiform Appendix Diet A; VA.B = Vermiform Appendix Diet B.

LEfSe analysis of the SR samples revealed that the phylum Bacteroidetes, the class Bacteroidia, the order Bacteroidales and the family Oxalabacteraceae were significantly enriched in animals submitted to diet A (Fig. 3a). In the SR of the animals on diet B, the only significantly overrepresented taxon was the family Dehalobacteriaceae (Fig. 3a). In the VA samples, several bacterial taxa were overrepresented in diet A compared with diet B. These included the family Lachnospiraceae, the order Clostridiales and the genus Dorea (Fig. 3b). Among the taxa overrepresented in the VA samples of animals submitted to diet B, family Odoribacteraceae and genus Butyricimonas were found (Fig. 3b).

### GALT cell composition analysis

**Figure 4:**
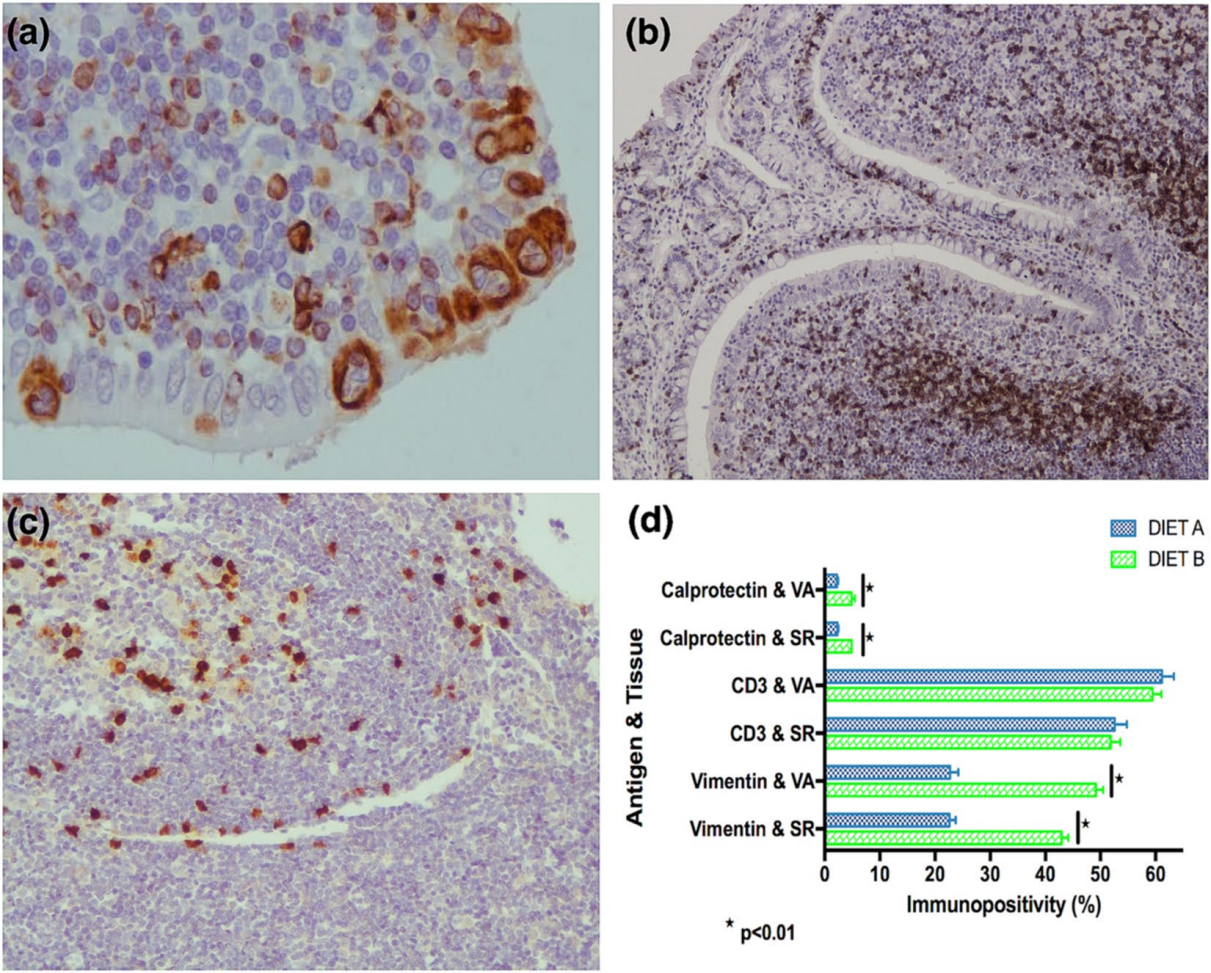
GALT Immunostaining of (**a**)Vimentin (**b**) CD3 and (**c**) Calprotectin antigens. (**d**) Immunohistochemical analysis of the Sacculus Rotundus (SR) and Vermiform Appendix (VA) samples of animals on diet A (DIET A) and diet B (DIET B). Error bars indicate standard error of the mean (SEM).

Immunohistochemistry analysis revealed that the percentage of vimentin positively immunostained cells in the epithelium of the dome was increased on diet B in both analyzed tissues, SR (p < 0.001) and VA (p < 0.001) (Fig. 4). Animals on diet B also presented a higher percentage of cells immunolabelled for calprotectin (MAC387), with a morphology consistent with macrophages, compared to animals on diet A, in the follicular and interfollicular areas of the SR (p < 0.001) and VA (p < 0.001) (Fig. 4). Regarding CD3 expression, the percentage of positively immunostained lymphocytes in the follicular and interfollicular area of the lymphoid tissue for this marker did not show significant differences between the analyzed diets neither in the SR (p = 0.635) or VA (p = 0.321) (Fig. 4).

### Microbiota and cell composition correlation analysis

**Figure 5:**
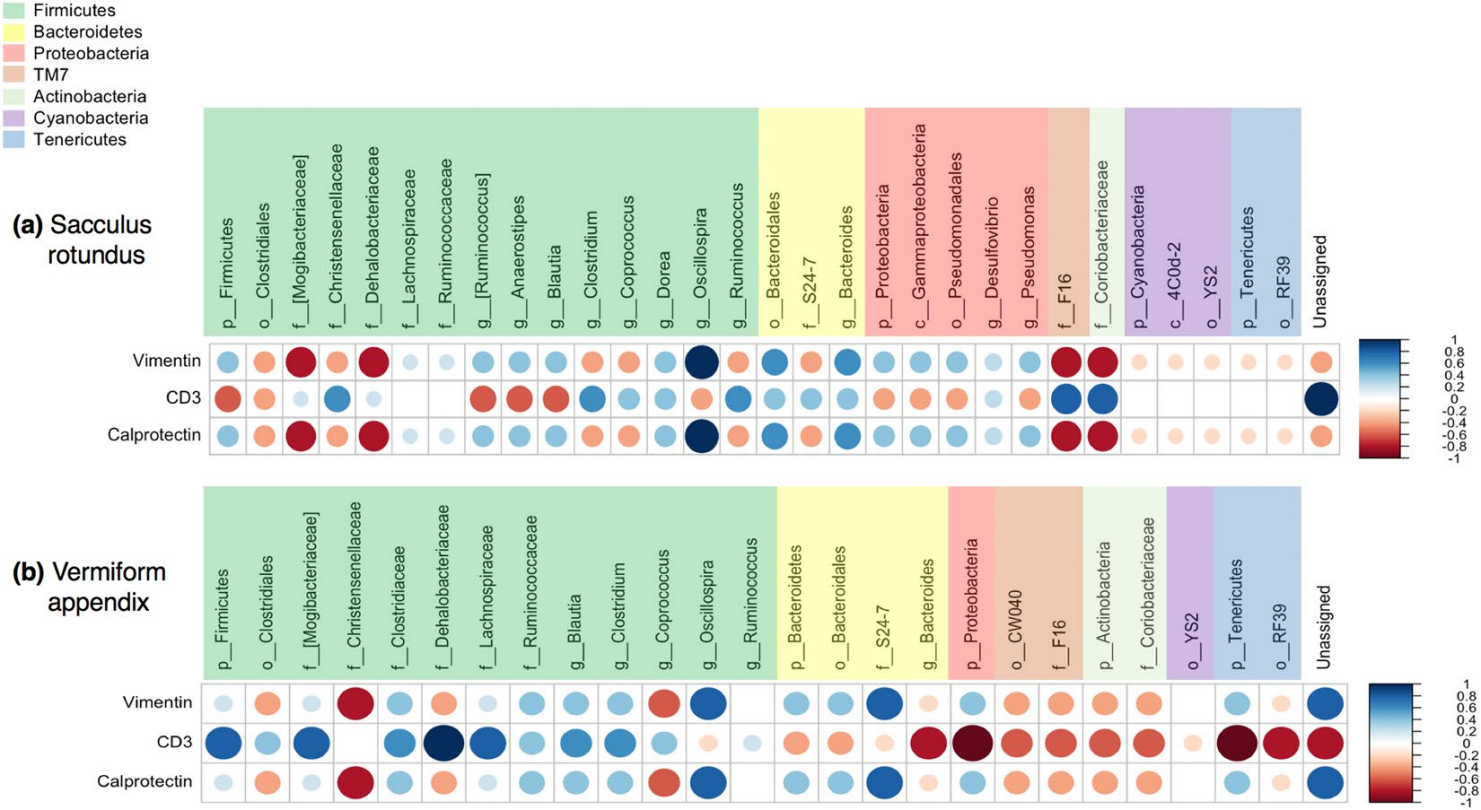
Correlations between microbiota (operational taxonomic unit with relative abundance higher than 0.1% of community) and immunopositivity of cell markers vimentin, CD3 and calprotectin in **(a)** sacculus rotundus and **(b)** vermiform appendix. Positive correlations are displayed in blue and negative correlations in red color. Color intensity and the size of the circle are proportional to the correlation coefficients.

Correlations between microbiota and immunopositivity of cell markers vimentin, CD3 and calprotectin per tissue are shown in Fig. 5. The phylum Firmicutes and families Mogibacteriaceae, Dehalobacteriaceae and Lachnospiraceae were positively correlated to marker CD3 in VA, whereas families F16 and Coriobacteriaceae were positively correlated to the same marker in SR. The genus *Oscillospora* was positively correlated to vimentin and calprotectin in both tissues.

Families Mogibacteriaceae, Dehalobacteriaceae, F16 and Coriobacteriaceae were negatively correlated to markers vimentin and calprotectin in SR, whereas these markers were negatively correlated with family Chistensenelleaceae and the genus *Coprococcus* in VA. In the VA there was a negative correlation of marker CD3 and the phyla Proteobacteria, Tenericutes and the genus *Bacteroides*.

In SR the unassigned microbial species were positively correlated with marker CD3 whereas in VA they were positively correlated with vimentin and calprotectin and negatively correlated with CD3.

## Discussion

Advances in the high-throughput sequencing technology over the last decade made it possible to thoroughly study the gut microbiota of rabbits^26–29^. Most of these studies have been carried out in feces and cecum and little is known about the microbiota colonizing the lymphoid tissue of specialized intestinal organs. The intestinal microbiota exists in reciprocal balance with the gut-associated lymphoid tissue (GALT), the largest immune organ in the body. Studies using a germ-free ligated rabbit VA show that specific bacteria can establish inflammatory responses in the gut^10^. The microbiota of the SR has been recently studied in a paratuberculosis infection model^30^ but this is the first study describing the microbiota of VA of rabbits.

Animals belonging to diet A showed lower weight values at the beginning of the experiment due to random assigning. Lower weight increase in Diet B animals was recorded only in the intervals 20–24 and 24–27 weeks concurring with the second diet shift. This was probably due to the low energy characteristics of this diet. In any case, this effect was reverted afterwards and total weight did not show significant differences among groups.

It has been reported that a high fibre diet increases microbial diversity at the gastrointestinal level^17^. However, in the present study changes in the alpha diversity indices at lymphoid tissue level were not observed when animals were on a high fibre diet, which included two periods of wheat straw diet intake (diet B). This finding could be related to the robustness of lymphoid tissue microbiota, meaning that it can resist short-term variation imposed by the transient gut microbiota without significant change in its diversity.

The diversity analyses between animals (beta-diversity) revealed that the microbial composition between diets A and B varied using both weighted and unweighted UniFrac distances, suggesting that diet impacted the presence and/or absence of certain taxa as well as their abundance. However, housing in separate rooms to avoid mixing diets and cecotrophy must be considered as it may have influenced the results. Although the effect of housing on rabbit microbiota composition has not been studied, it could have an impact, similarly to the commonly known as “cage effect” described in mice^31^.

Microbiota of rabbit digestive specialized lymphoid organs was dominated by the phylum Firmicutes as reported recently^30^. This phylum is predominant not only in hindgut fermenters like rabbits and horses, but also in ruminants and monogastric animals^32^.

Contrary to what has been observed in rabbits cecal content^26,27^ or feces^28,29^ the second predominant phylum was TM7. The candidate phylum TM7 is composed of non culturable and highly ubiquitous bacteria^33^. It has also been associated with human diseases such as periodontitis^34^ and inflammatory bowel disease^35^. The deficiency of NOD-like receptor P6 inflammasome in mouse colonic epithelial cells resulted in the alteration of fecal microbiota with the overrepresentation of TM7 and Bacteroidetes^36^, suggesting that the abundance of these phyla are modulated by the immune system. Therefore, the high presence of this phylum in the lymphoid organs could be related to the immune nature of the tissue, and it could induce the necessary stimulation to activate the mechanisms for the appropriate performance of the immune system.

Among animals on diet B, two taxa of the phylum Bacteroidetes (family Dehalobacteriaceae and genus *Butyricimonas*) were overrepresented. The phylum Bacteroidetes is a very diverse bacterial phylum that has colonized many different ecological environments, including soil, ocean, freshwater, and the gastrointestinal tract of animals^37^. This phylum, has a major role in degrading complex polysaccharides^38^ that are normally present in high fibre diets. Gut Bacteroidetes generally produce butyrate, an end product of colonic fermentation, which is thought to have antineoplastic properties and thus plays a role in maintaining a healthy gut^39^. Studies have shown that high fibre based diets promote the abundance of Bacteroidetes in rabbits gut^27,40^. This finding is compatible with our results because animals submitted to diet B were fed wheat straw, characterized by its high fibre content. Diet B is high in fibre but also low in fat and protein and this cannot be dismissed. In a previous study it has been shown that moderate dietary protein restriction optimized gut microbiota and mucosal barrier in growing pig model^41^. In addition, a high-fat diet was also associated with a decrease in bacterial richness and bacterial taxa known to produce the short chain fatty acid butyrate^42^. However, low fat diet has not been associated with changes in the microbiota composition over time^43^.

Vimentin immunostaining has been widely used to detect rabbit M cells in the GALT as this protein is part of the cytoskeleton and it is not expressed in other cells present in the lining epithelium such as enterocytes or lymphocytes^24,44^. In the present study, animals under diet B presented higher percentage of vimentin immunopositive cells in the epithelium of both analyzed lymphoid tissues, indicating that dietary fibre could have raised the abundance of M cells in these tissues. In previous studies, also performed in rabbits, high fibre/low starch diet has shown to increase M cell size and number^24^.

An interesting finding of this study is that animals on the diet B also presented higher numbers of cells positively immunostained for calprotectin in both analyzed lymphoid tissues. Calprotectin is a major cytosolic protein complex present in monocytes that is expressed in tissue macrophages recently recruited from peripheral blood^45^. This could be a consequence of the increase in the number of M cells, since M cell hyperplasia may lead to the penetration of a higher number of microorganisms, resulting in the activation of blood monocytes and the migration of these to the lymphoid tissue.

Bacteroidetes interact with the immune system of the host activating T-cell mediated responses^46^. In this study, no differences in the percentage of T cells (CD3 immunostaining) were observed between both diets meaning that T-cell levels were maintained. This could be due to the fact that the activation of T-cell mediated immunity is not associated with a greater number of T cells but to more active T cells. However, it could also be due to the application of a technique that is not capable of detecting small changes in cellular composition that could have been identified with other technologies that analyze a higher number of cells (for example fluorescence-activated cell sorting).

Surprisingly, in this study, the phylum Bacteroidetes has also been detected in higher percentage in the SR of animals submitted to diet A. These findings point out that it is necessary to develop more studies with a shotgun metagenomic or metatranscriptomic approach combined with immunological assays that could provide more information about the role of this relevant phylum in gut immunity. In addition, in the SR of animals on the diet A, a member of the phylum Proteobacteria has been detected overrepresented (family Oxalabacteriaceae). Proteobacteria are considered to be minor and opportunistic components of the gut ecosystem and they have been associated with inflammatory bowel disease^47^. Moreover, the abundance of Oxalobacteraceae has been negatively correlated with the expression of IFNγ and TLR10^48^ suggesting that the overrepresentation of this taxa could be related to the lower fibre diet intake or to the less active immunity.

Several taxa of the phylum Firmicutes have been identified overrepresented in the VA of animals on diet A (order Clostridiales, family Lachnospiraceae, and genus *Dorea*). The family Lachnospiraceae is one of the most abundant families from the order Clostridiales found in the mammalian gut environment, and have been associated with the maintenance of gut health^49^. Nevertheless, the results of other studies raise doubts about its benefits. In mice treated with low doses of penicillin V the overrepresentation of the Lachnospiaraceae in stool samples has been described^50^. In addition, the increased relative abundance of this family has been observed at weaning as a predictor of diabetes and immune status later in life^51^. The genus *Dorea*, was underrepresented among subjects with food allergy^52^ suggesting that it may promote or protect against food sensitization and food allergy. However, this genus has also been identified as a harmful bacteria because it has been detected in high levels in patients with amyotrophic lateral sclerosis (ALS)^53^. The presence of these Firmicutes taxa underrepresented in the VA of animals submitted to diet B could be a direct effect of the dietary intake. In spite of that, this finding may be a consequence of the effects of diet on the immune system which could have modulated the gut ecosystem.

When correlating cell composition and microbiota some differences were observed among tissues, as the opposite correlation of the phylum Firmicutes with CD3 marker depending on the tissue (negative correlation in SR and positive in VA). These differences could be related to the localization of the tissue in the digestive system (the beginning or the end of the cecum) and it could imply that some microbial taxa could promote different effects depending on the location across the digestive system. The genus *Oscillospora* has been positively correlated to vimentin and calprotectin in both tissues. This genus has been associated with leanness and health in humans and mice^54^.

Summarizing, in the present study changes in the microbiota and in the lymphoid tissue cell composition were detected in relationship with changes in the nutritional content of the diet in a small number of animals. Even though it is not possible to determine if the changes in the microbiota are the cause or the consequence of the changes observed in the lymphoid tissue cell composition, it is clear that there is an interaction between the microbiota and the immunity in the GALT. Further work may include validation in a larger number of animals or studies focused on the identification of the connection pathways between the microbiota and immunity; all leading to prevention or treatment strategies of several diseases through the diet and/or microbiota modifications.

## Materials and Methods

### Animals and experimental designs

New Zealand white female rabbits (n = 10) were purchased from authorized experimental animals dealers (Granja San Bernardo, Tulebras, Spain) and arrived at NEIKER animal facilities at the age of 6 weeks. After a two-week adaptation period during which rabbits were fed weaning pellets, all animals started taking a regular commercial growing diet containing neither antibiotics, nor coccidiostatics. Feeding was limited to 30–35 g/day of dry matter/kg of live weight throughout the experiment whereas water was available *ad libitum*.

After adaptation, two different diets were assayed following the experimental scheme detailed on Supplementary Fig. S1. Five animals were randomly assigned to diet A or control, being fed the commercial diet pellets. The remaining 5 animals were assigned to diet B in which the commercial diet was switched to a wheat straw pellet diet for two weeks at two different time points: week seven and twenty-three. Following each of these periods, diet was switched back to the commercial diet in a stepwise manner by replacing 25% of diet weekly, returning to regular diet at week 12 and 28.

Both groups of animals were housed in separate rooms to avoid mixing diets and cecotrophy between animals of the different diet groups. Animals were weighed at timepoints 0, 7, 11, 16, 20, 24, 27 and 31 weeks. All animals were euthanized at week 31 (age 39 weeks) by intracardiac pentobarbital injection after deep sedation with xylazine (5 mg/kg) and ketamine (35 mg/kg).

### Microbiota analysis

#### Sampling and DNA extraction for 16S rRNA gene sequencing

The SR and VA were excised and the mucosa was carefully scraped to remove the digesta, following by a vigorous wash with sterile phosphate-buffered saline (PBS). A fragment was divided and saved immediately in a sterile microtube. All samples were stored in liquid nitrogen until further processing.

DNA extraction was carried out with Ultra-Deep Microbiome Prep kit (Molzym, Bremen, Germany) following manufacturer’s instructions. This method enriches bacterial DNA and removes host and “dead” microbial DNA. Briefly, 0.25 cm^2^ of tissue was treated for host cell lysis with PKB buffer and proteinase K following a treatment with a chaotropic buffer that lyses animal cells (Buffer CM). The host and dead bacteria released DNA was enzymatically degraded (MolDNaseB) and after that the degrading enzymes were inactivated (buffer RS). In the last step the DNA of remaining whole cell bacteria was extracted. Bacterial cell wall was degraded (BugLysis solution and proteinase K), and finally the DNA was extracted and purified via silica based spin columns. DNA concentration and purity was determined with ND-1000 spectrophotometer (Nanodrop, Wilmington, DE, USA) by measuring the A _260/280_. DNA quality was evaluated by gel electrophoresis after standard PCR using universal primers pAF (5′-AGA GTT TGA TCC TGG CTC AG-3′) and 530 R (5′-CCG CGG CKG CTG GCAC-3′). DNA extracts were stored at −20 °C until they were processed.

#### Library construction and MiSeq Illumina sequencing

The V3-V4 region of 16S rRNA gene was targeted for PCR amplification using a modified F338 and barcoded R806 primers as described previously^55^. Briefly, PCR reaction for each sample was performed in duplicate and contained 1.0 µl of pre-normalized DNA, 1.0 µl of each forward and reverse primers (10 µM), 12 µl HPLC grade water (Fisher Scientific, ON, Ottawa, Canada) and 10 µl 5 Prime Hot MasterMix (5 Prime, Inc., Gaithersburg, MD, USA). Reactions consisted of an initial denaturing step at 94 °C for 3 min followed by 30 amplification cycles at 94 °C for 45 sec, 62 °C for 60 sec, and 72 °C for 90 sec; finalized by an extension step at 72 °C for 10 min. PCR products were purified using ZR-96 DNA Clean-up Kit™ (Zymo Research, Irvine, CA, USA). The V3-V4 libraries were then generated by pooling 200 ng of each sample, quantified by Picogreen dsDNA (Invitrogen, Burlington, On, Canada) and diluted to a final concentration of 5 pM, measured by Qubit 2.0 Fluorometer (Life technologies, Burlington, ON, Canada). In order to improve the unbalanced and biased base composition of the generated 16S rRNA libraries, 15% of PhiX control library was spiked into each amplicon pool. Customized sequencing primers for read1 (5′-TATGGTAATTGTGTGCCAGCMGCCGCGGTAA-3′), read2 (5′-AGTCAGTCAGCCGGACTACHVGGGTWTCTAAT-3′) and index read (5′-ATTAGAWACCCBDGTAGTCCGGCTGACTGACT-3′) were synthesized and purified by polyacrylamide gel electrophoresis (Integrated DNA Technologies, Coralville, IA, USA) and added to the MiSeq Reagent Kit V3 (600-cycle) (Illumina, San Diego, CA, USA). The 300 paired-end sequencing reactions were performed on a MiSeq platform (Illumina, San Diego, CA, USA) at the Gut Microbiome and Large Animal Biosecurity Laboratories, Department of Animal Science, University of Manitoba, Canada.

#### Data deposition

The 16S rRNA profiling data were deposited into the Sequence Read Archive (SRA) database of NCBI (http://www.ncbi.nlm.nih.gov/sra) and can be accessed via SRR2962702 accession number.

#### Bioinformatics and statistical analyses

The FLASH assembler^56^ was used to merge overlapping paired-end Illumina fastq files. All the sequences with mismatches or ambiguous calls in the overlapping region were discarded. The output fastq file was then analyzed by downstream computational pipelines of the open source software package QIIME version 1.9.0^57^. Assembled reads were demultiplexed and exposed to additional quality-filters so that reads with ambiguous calls and those with phred quality scores (Q-scores) below 20 were discarded. Chimeric reads were filtered using UCHIME^58^ and sequences were assigned to Operational Taxonomic Units (OTU) using UCLUST^59^ at 97% pairwise identity threshold. Taxonomies assignment of representative OTUs and alignment to Silva reference database were performed using PyNAST algorithms^60^.

Within community diversity (alpha-diversity) was calculated using QIIME scripts. An even depth of 12,400 sequences per sample was used for calculation of species richness (Chao1) and diversity indices (Shannon and Simpson) for the SR and VA, respectively. One of the animals did not reach the minimum number of reads to be included in the downstream analysis and was removed from the microbiota analysis. Alpha rarefaction curves were generated using observed species (richness) with ten sampling repetitions at each sampling depth. R software was used to test the normality of residuals for alpha-diversity indices and the average of bacterial phyla. A logarithmic transformation was used to normalize the data when necessary. Comparisons between groups were performed using Student’s t-test.

The diversity between animals and treatments (beta-diversity) was compared using weighted and unweighted UniFrac distances^61^ based on phylogenetic differences. Principal coordinate analysis (PCoA) was applied on resulting distance matrices to generate two-dimensional plots using PRIMER V6 software^62^ and permutational multivariate analysis of variance (PERMANOVA) was used to calculate *p*-values and test for differences between microbial communities.

Microbial community differences between diets were analyzed through the linear discriminant analysis (LDA) effect size (LEfSe) in order to identify taxa that were discriminant between the two clusters^63^. For this analysis, a table of taxa abundance including all the different taxonomic levels was used. Briefly, the first step of the LEfSe method analyzed all taxonomic units, testing whether abundance in the different clusters (designed as classes by the LEfSe method) are differentially distributed, using a Kruskal-Wallis rank sum test. An LDA model was then built to estimate the effect size of each differentially abundant taxon. This step resulted in a list of taxonomic units that are discriminative with respect to the classes. A *p*-value of <0.05 was considered to be statistically significant.

### Immunohistochemical study

#### Immunohistochemistry

Samples from the SR and VA were collected and fixed in 10% neutral buffered formalin solution for a minimum of 24 h, trimmed and dehydrated through graded alcohols. After, samples were embedded in paraffin and sectioned at 3–5 μm. Sections were placed on poly-L-lysine slides and immunohistochemically stained using the Envision + System (Dako, Agilent Technologies, Glostrup, Denmark). Different monoclonal antibodies raised against antigens expressed in M cells, activated macrophages and T lymphocytes were used (Supplementary Table 1).

Briefly, sections were deparaffinized and antigen retrieval was performed in the PT Link system (Dako, Agilent Technologies, Glostrup, Denmark) at 96 °C using a pH 6 or pH 9 retrieval solution (Supplementary Table 1). After hydration, the sections were incubated in 3% hydrogen peroxide in methanol for 30 min to eliminate endogenous peroxidase. Rehydrated slides were rinsed in PBS of pH 7.4, and sections were incubated with the primary antibodies diluted in PBS (Supplementary Table 1) overnight at 4 °C in a humidified chamber. After washing in PBS, sections were incubated for 40 minutes at room temperature with EnVision + horseradish peroxidase solution (Dako, Agilent technologies) for the appropriate monoclonal antibodies. After washing in PBS, antibody localization was determined using 3,3-diaminobenzidine (Sigma-Aldrich Corp., Madrid, Spain). Sections were counterstained with Mayer’s hematoxylin for 10 seconds. Slides were mounted with DPX (dibutyl phthalate xylene) and observed under a light microscope. Appropriate species and isotype-matched immunoglobulins were used as negative controls.

#### Image analysis and statistics

In each slide, 20 representative fields were selected, photographed and analyzed with image J software. In each image, the area for the analysis was selected. For vimentin, only positively immunolabelled cells present in the dome epithelium or follicle associated epithelium were selected and for CD3 and Calprotectin positively immunostained cells were counted in the follicular and interfollicular area of the lymphoid tissue. Then, the images were analyzed using IHC profiler plugin^64^, which creates a pixel-by-pixel analysis profile of the digital image. The percentage of positivity in the selected area was calculated by the sum of the percentage of high positive pixels (intensity range, 0–60) and the percentage of positive pixels (intensity range, 61–120) given during the IHQ profiler analysis.

Since the data were not normally distributed, statistical comparison between groups was performed using the non-parametric Mann-Whitney-Wilcoxon test implemented in R software and a p-value of <0.05 was considered to be statistically significant.

Non-parametric Spearman rank correlation analysis was used to test the relationship between cell composition and the bacterial communities in SR and VA (OTUS with more than 0.1% of relative abundance). The resulting correlation matrix was visualized using corrplot package implemented in R (version 3.4.4) software.

The datasets generated during and/or analysed during the current study are available from the corresponding author on reasonable request.

### Ethics statement

This study was carried out following European, National and Regional regulations on animals used in experimentation and other scientific purposes. The procedure was evaluated by the Ethics Committee at NEIKER (NEIKER-OEBA-2014-0001) and authorized by the Regional Council (BFA-4269).

## Electronic supplementary material

Supplementary information
