# Supplementary material for: Diet induced changes in the microbiota and cell composition of rabbit gut associated lymphoid tissue (GALT)
Source: Sci Rep. 2018 Sep 20;8:14103. doi: 10.1038/s41598-018-32484-1 (PMC6148544; doi:10.1038/s41598-018-32484-1)

Diet induced changes in the microbiota and cell composition of rabbit gut associated lymphoid tissue (GALT)

Rakel Arrazuria[1]. Valentín Pérez[2]. Elena Molina[1]. Ramón A. Juste[1^]. Ehsan Khafipour [3,4]. Natalia Elguezabal[1]*

[1]Department of Animal Health. NEIKER-Instituto Vasco de Investigación y Desarrollo Agrario. Derio. Bizkaia. Spain
[2]Department of Animal Health. Faculty of Veterinary Medicine. University of Leon. Leon. Spain.
[3]Department of Animal Science. University of Manitoba. Winnipeg. MB. Canada 10
[4]Department of Medical Microbiology. University of Manitoba. Winnipeg. MB. Canada
^Current address: SERIDA. Agri-food Research and Development Regional Service. Villaviciosa. Asturias. Spain

Supplementary Table 1. Details of the primary antibodies and procedures used in the immunohistochemistry.

Antigen (Clone)	Target	Specifity	Epitope demasking	Dilution
Calprotectin (MAC 387)	Myeloid Histiocyte	Circulating (and emigrated) neutrophils and monocytes. as well as a subset of reactive tissue macrophages recently recruited.	Heat mediated AR. EDTA buffer. pH 9	1/200
Vimentin (V9)	Mesenchymal cells	M cells of rabbit gut-associated lymphoid tissue (protein of the cytoskeleton) [1]. Highly expressed in fibroblasts. some expression in T- and B-lymphocytes.	Heat mediated AR. Citrate buffer. pH 6	1/100
CD3 (F7.2.38)	T cells	T cells. thymocytes. and activated natural killer cells	Heat mediated AR. Citrate buffer. pH 6	1/200

AR: Antigen Retrieval

Supplementary Table 2. Animal weight parameters

(a) Weight at different experimental periods

Diet		Time (weeks)							
		0	7	11	16	20	24	27	31
A	Mean	1.769	2.756	3.099	3.510	3.801	4.143	4.344	4.463
	SEM	0.061	0.0449	0.1083	0.1340	0.1306	0.1398	0.1517	0.1462
B	Mean	2.230	3.038	3.457	3.885	4.232	4.518	4.489	4.620
	SEM	0.1109	0.0982	0.1246	0.1941	0.2175	0.0292	0.2225	0.2194
	p	0.07*	0.031*	0.062*	0.151	0.128	0.280	0.605	0.568

(b) Mean weight increase along different experimental periods.

Diet		Time intervals (weeks)							
		w7-w0	w11-w7	w11-w0	w24-w20	w27-w24	w27-w20	w31-w27	w31-w0
A	Mean	0.987	0.343	1.330	0.342	0.201	0.543	0.119	2.694
	SEM	0.0566	0.0943	0.1436	0.1146	0.0334	0.0958	0.0099	0.1832
B	Mean	0.808	0.419	1.227	0.286	-0.029	0.257	0.131	2.390
	SEM	0.0915	0.0879	0.1338	0.0897	0.0767	0.0194	0.0146	0.2658
	p	0.135	0.572	0.614	0.711	0.025*	0.019*	0.516	0.374

SEM: standard error of the mean. w: week. *p*: p values of Student T-test. *significant differences among groups.

Supplementary Figure 1. Experimental design **(a)** and post-mortem sampling **(b)**.

(a) Experimental design

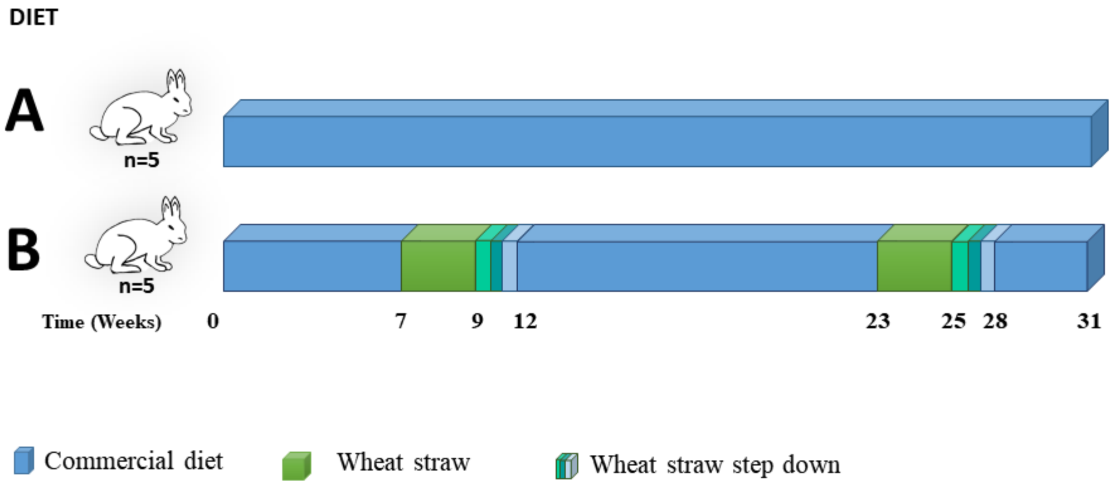

Nutrient composition: Commercial diet (90.26% of dry matter, 16.3% of crude protein, 3.04% of crude fat, 14.36% of crude fibre and 7.04% of crude ash) and wheat straw (93.3% of dry matter, 2.69% of crude protein, 1.02% of crude fat, 35.05% of crude fibre and 8.40% of crude ash).

(b) Post-mortem sampling organs

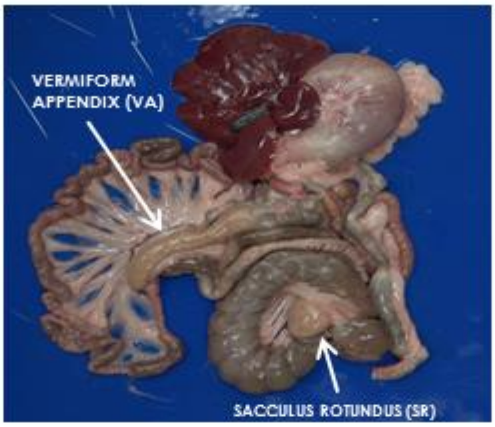

Supplementary Figure 2. Mean weight of diet groups at different time periods. Error bars indicate standard error of the mean (SEM).

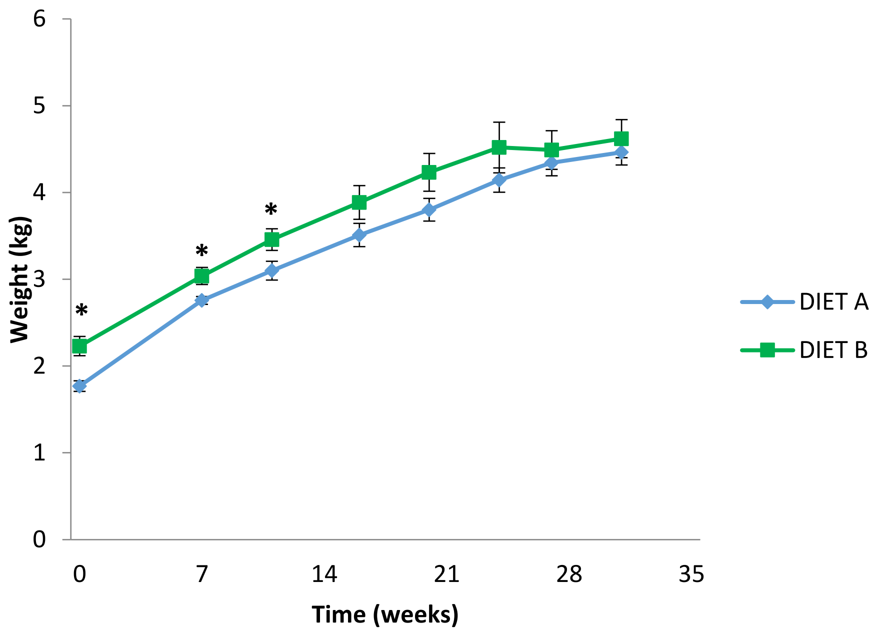

*significant differences among groups p<0,05

Supplementary Figure 3. Lymphoid tissue microbiota rarefaction curve generated using Observed species (richness). Samples have been rarified at an even depth of 12.400 sequences per sample. Error bars indicate the 95% confidence intervals. SR_A= Sacculus Rotundus Diet A; SR_B= Sacculus Rotundus Diet B; VA_A= Vermiform Appendix Diet A; VA_B= Vermiform Appendix Diet B.

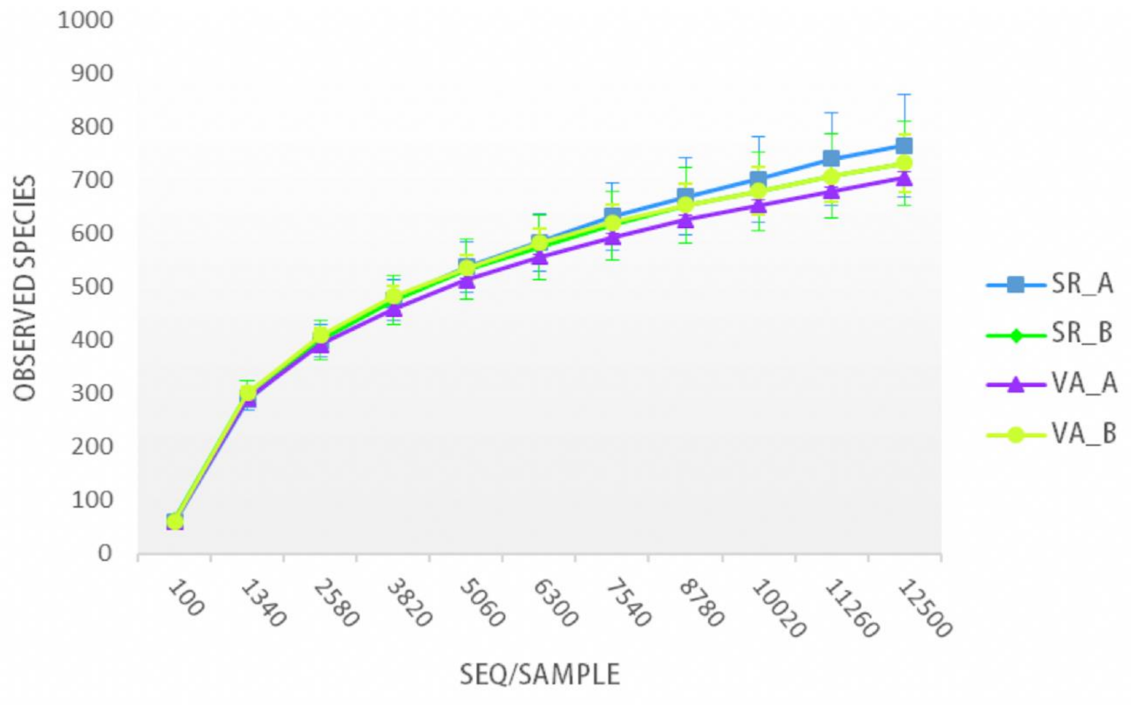

Supplementary Figure 4. Sacculus Rotundus and Vermiform Appendix alpha-diversity; richness(Chao1) and diversity (Shannon and Simpson) indices boxplot.

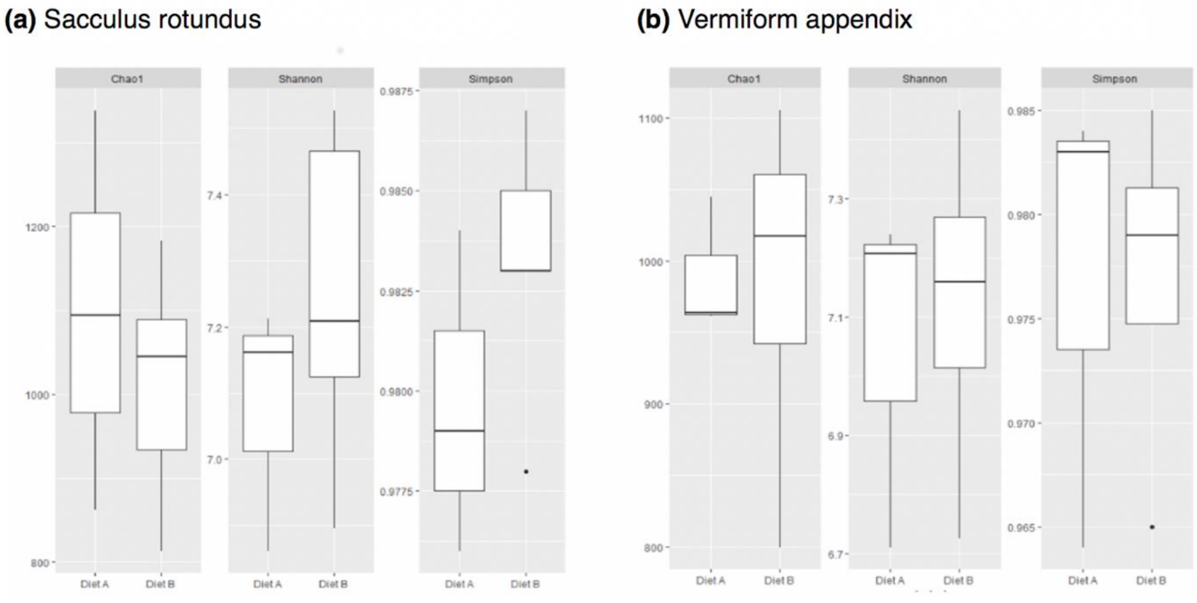

Supplement: Supplementary file 1 — Supplementary information [file 41598_2018_32484_MOESM1_ESM.pdf]
